# Supplementary material for: Mortality Prediction Modeling for Patients with Breast Cancer Based on Explainable Machine Learning
Source: Cancers (Basel). 2024 Nov 12;16(22):3799. doi: 10.3390/cancers16223799 (PMC11592669; doi:10.3390/cancers16223799)
Supplement: Supplementary file 1 [file cancers-16-03799-s001.zip › Supplement_tables.pdf]

Table S1. All features from hospital registry database

Health and demographical information	Laboratory results	Treatment	Pathology	Others
Age at diagnosis	WBC	Radiation treatment for curative	p53 (%)	Occurrence in other organs
Height	Fasting glucose	Chemotherapy	Ki-67 (%)	Tumor in bilateral
BMI	Total cholesterol	Anti HER2	Tumor subtype	Tumor location
Smoking	Date of mammogram examination	Anti hormone	T stage	Tumor location index
Drinking	Mammographic density	Surgical operation type	N stage	
Age at menarche		BSO status	Histology grade	
Age at menopause		Date of BSO surgery	Estrogen receptor test result	
Parturition experience		Hysterectomy status	Progesterone receptor test result	
Experience of oral contraceptives		Purpose of radiation treatment	Androgen receptor test result	
Hormone replacement therapy		Radiation dose per fraction	HER2 score	
Family history		Hormone therapy prescription date	p53 positive/negative, equivocal	
Parents cancer history		Hormone therapy medication name	Ki-67 positive/negative	
Cancer history		Neoadjuvant chemotherapy status	Tumor size	
Date of death		Adjuvant chemotherapy status	BRCA1 mutation code	
Date of initial diagnosis		Chemotherapy, hormone therapy, targeted therapy administration status	Unclassified BRCA1 variant code	
Date of diagnosis			BRCA2 mutation code	
Weight			Unclassified BRCA2 variant code	
Pregnancy at breast cancer diagnosis				
Education level				
Occupation				
Menopausal status				
Date of last follow-up				
Follow-up period				

BMI, body mass index; WBC, white blood cell; HER2, human epidermal growth factor receptor 2; BSO, bilateral salpingo-oophorectomy; BRCA, breast cancer gene.

Table S2. Primary features from hospital registry database

Health and demographical information	Laboratory results	Treatment	Pathology	Others
Age at diagnosis	WBC	Radiation treatment for curative	p53 (%)	Occurrence in other organs
Height	Fasting glucose	Chemotherapy	Ki-67 (%)	Tumor in bilateral
BMI	Total cholesterol	Anti HER2	Tumor subtype	Tumor location
Smoking		Anti hormone	T stage	Tumor location index
Drinking		Surgical operation type	N stage	
Age at menarche			Histology grade	
Age at menopause				
Parturition experience				
Experience of oral contraceptives				
Hormone replacement therapy				
Family history				
Parents cancer history				
Cancer history				

BMI, body mass index; WBC, white blood cell; HER2, human epidermal growth factor receptor 2.

Table S3. Details of imputed feature information

Feature	Number of missing	Imputed value - mean	Imputed value - mode
Height	11	157.35	
BMI	11	28.83	
Drinking	1		0 (No)
Smoking	1		0 (No)
Cancer history	406		0 (No)
Age at menarche	1,880	14.79	
Age at menopause	6,428	49.51	
Experience of oral contraceptives	708		0 (No)
Hormone replacement therapy	708		0 (No)
Family history	708		0 (No)
Parturition experience	708		1 (Yes)
Parents cancer history	2,179		3 (None)
p53 (%)	3,914	28.55	
Ki-67 (%)	703	26.14	
Histology grade	316		pathology reports data (nuclear grade), mode (2)
T stage	498		pathology reports data (p stage, t size), mode (1)
N stage	540		pathology reports data (p stage), mode (0)

BMI, body mass index.

Table S4. Standardized mean differences before and after PSM in each group

	Overall breast cancer group		Breast cancer-only group	
	**Before PSM**	**After PSM**	**Before PSM**	**After PSM**
**Age at diagnosis**	0.35	0.36	0.52	0.51
**Height**	-0.31	-0.32	-0.37	-0.35
**BMI**	0.14	0.14	0.18	0.18
**Smoking**				
No	0.03	0.03	0.01	0.01
Yes	0.03	0.03	0.01	0.01
**Drinking**				
No	0.09	0.09	0.10	0.12
Yes	0.09	0.09	0.10	0.12
**Age at menarche**	0.23	0.23	0.30	0.25
**Age at menopause**	-0.08	-0.08	-0.06	-0.06
**Parturition experience**				
No	0.01	0.01	0.00	0.00
Yes	0.01	0.01	0.00	0.00
**Experience of oral contraceptives**				
No	0.01	0.01	0.00	0.00
Yes	0.01	0.01	0.00	0.00
**Hormone replacement therapy**				
No	0.01	0.02	0.01	0.01
Yes	0.01	0.02	0.01	0.01
**Family history**				
No	0.04	0.04	0.05	0.06
Yes	0.04	0.04	0.05	0.06
**Parents cancer history**				
Paternity	0.05	0.05	0.05	0.04
Maternal line	0.05	0.05	0.05	0.07
Parental	0.02	0.01	0.02	0.02
None	0.11	0.11	0.12	0.12
**Cancer history**				
No	0.01	0.00	0.02	0.00
Yes	0.01	0.00	0.02	0.00
**Total cholesterol**	-0.02	-0.02	0.03	0.05
**Fasting glucose**	0.30	0.28	0.34	0.31
**WBC**	0.18	0.22	0.19	0.19
**Surgical type**				
None	0.00	0.00	0.00	0.00
BCS	0.13	0.13	0.14	0.13
Mastectomy	0.13	0.14	0.14	0.13
**Tumor location**				
Left	0.02	0.02	0.05	0.05
Right	0.02	0.02	0.03	0.03
Both	0.01	0.00	0.02	0.02
**Tumor location index**				
Single	0.01	0.00	0.02	0.02
Bilateral & synchronous	0.00	0.01	0.01	0.01
Bilateral & metachronous	0.01	0.00	0.01	0.01
**Bilateral**				
No	0.01	0.01	0.01	0.01
Yes	0.01	0.01	0.01	0.01
**Occurrence in other organs**				
No	0.31	0.31		

	Yes	0.31	0.31		
**Histologic grade**					
	1	0.07	0.05	0.06	0.04
	2	0.14	0.14	0.14	0.14
	3	0.21	0.20	0.20	0.18
**p53(%)**		0.21	0.18	0.20	0.18
**Ki-67(%)**		0.33	0.33	0.32	0.25
**T stage**					
	0	0.03	0.03	0.03	0.02
	1	0.24	0.22	0.26	0.25
	2	0.17	0.15	0.18	0.17
	3	0.07	0.07	0.06	0.06
	4	0.04	0.03	0.05	0.05
**N stage**					
	0	0.29	0.29	0.28	0.27
	1	0.09	0.10	0.10	0.09
	2	0.12	0.12	0.11	0.11
	3	0.07	0.07	0.07	0.07
**Tumor subtype**					
	Luminal A	0.06	0.05	0.08	0.08
	Luminal B	0.01	0.01	0.00	0.00
	Basal	0.06	0.05	0.09	0.09
	HER2 overexpressing	0.01	0.01	0.01	0.02
**Radiation treatment for curative**					
	Yes	0.16	0.16	0.19	0.18
	No	0.16	0.16	0.19	0.18
**Chemotherapy**					
	None	0.01	0.01	0.01	0.01
	Adjuvant	0.20	0.20	0.18	0.18
	Neoadjuvant	0.20	0.20	0.15	0.15
**Anti HER2**					
	No	0.16	0.16	0.08	0.08
	Yes	0.16	0.16	0.08	0.08
**Anti hormone**					
	No	0.09	0.09	0.14	0.14
	Yes	0.09	0.09	0.14	0.14

PSM, propensity score matching; BMI, body mass index; WBC, white blood cell; BCS, breast conserving surgery; HER2, Human epidermal growth factor receptor 2.

Table S5. Participant characteristics before propensity score matching

	Overall breast cancer group				Breast cancer-only group			
	Total (N=11,286)	Death (N=695)	Alive (N=10,591)	*P*	Total (N=10,215)	Death (N=426)	Alive (N=9,789)	*P*
**Age at diagnosis**	50.7±10.6	54.7±14.0	50.4±10.3	<0.001	50.7±10.6	57.0±14.6	50.4±10.3	<0.001
**Height**	157.4±5.7	155.6±6.2	157.5±5.6	<0.001	157.4±5.7	155.2±6.3	157.5±5.6	<0.001
**BMI**	23.8±3.5	24.3±3.6	23.8±3.5	<0.05	23.8±3.5	24.4±3.7	23.7±3.5	<0.05
**Smoking**				<0.05				0.419
No	10,664 (94.5)	638 (91.8)	10,026 (94.7)		9,664 (94.6)	397 (93.2)	9,267 (94.7)	
Yes	622 (5.5)	57 (8.2)	565 (5.3)		551 (5.4)	29 (6.8)	522 (5.3)	
**Drinking**				<0.001				<0.001
No	8,809 (78.1)	600 (86.3)	8,209 (77.5)		7,946 (77.8)	373 (87.6)	7,573 (77.4)	
Yes	2,477 (21.9)	95 (13.7)	2,382 (22.5)		2,269 (22.2)	53 (12.4)	2,216 (22.6)	
**Age at menarche**	14.8±1.5	15.1±1.5	14.8±1.5	<0.001	14.8±1.5	15.2±1.5	14.8±1.5	<0.001
**Age at menopause**	49.5±3.2	49.3±3.8	49.5±3.2	0.094	49.5±3.1	49.3±4.0	49.6±3.1	0.358
**Parturition experience**				0.905				0.985
No	1,442 (12.8)	85 (12.2)	1,357 (12.8)		1,299 (12.7)	53 (12.4)	1,246 (12.7)	
Yes	9,844 (87.2)	610 (87.8)	9,234 (87.2)		8,916 (87.3)	373 (87.6)	8,543 (87.3)	
**Experience of oral contraceptives**				0.910				0.971
No	10,107 (89.6)	619 (89.1)	9,488 (89.6)		9,148 (89.6)	380 (89.2)	8,768 (89.6)	
Yes	1,179 (10.4)	76 (10.9)	1,103 (10.4)		1,067 (10.4)	46 (10.8)	1,021 (10.4)	
**Hormone replacement therapy**				0.890				0.915
No	10,398 (92.1)	637 (91.7)	9,761 (92.2)		9,407 (92.1)	390 (91.5)	9,017 (92.1)	
Yes	888 (7.9)	58 (8.3)	830 (7.8)		808 (7.9)	36 (8.5)	772 (7.9)	
**Family history**				<0.001				<0.001
No	10,387 (92.0)	667 (96.0)	9,720 (91.8)		9,397 (92.0)	414 (97.2)	8,983 (91.8)	
Yes	899 (8.0)	28 (4.0)	871 (8.2)		818 (8.0)	12 (2.8)	806 (8.2)	
**Parents cancer history**				<0.001				<0.001
Paternity	1,131 (10.0)	40 (5.8)	1,091 (10.3)		1,047 (10.2)	23 (5.4)	1,024 (10.5)	
Maternal line	804 (7.1)	16 (2.3)	788 (7.4)		740 (7.2)	10 (2.3)	730 (7.5)	
Parental	261 (2.3)	6 (0.9)	255 (2.4)		236 (2.3)	3 (0.7)	233 (2.4)	
None	9,090 (80.5)	633 (91.1)	8,457 (79.9)		8,192 (80.2)	390 (91.5)	7,802 (79.7)	
**Cancer history**				0.685				0.201
No	10,755 (95.3)	667 (96.0)	10,088 (95.3)		9,775 (95.7)	415 (97.4)	9,360 (95.6)	
Yes	531 (4.7)	28 (4.0)	503 (4.7)		440 (4.3)	11 (2.6)	429 (4.4)	
**Total cholesterol**	195.0±36.2	194.3±39.2	195.0±36.0	0.870	195.3±36.2	196.4±40.2	195.2±36.1	0.812

**Fasting glucose**	110.4±33.2	122.8±54.4	109.6±31.1	<0.001	109.9±32.3	124.2±54.6	109.3±30.8	<0.001
**WBC**	6.4±2.2	6.8±2.5	6.4±2.2	<0.001	6.4±2.2	6.9±2.8	6.4±2.2	<0.001
**Surgical type**				<0.001				<0.001
None	170 (1.5)	9 (1.3)	161 (1.5)		156 (1.5)	5 (1.2)	151 (1.5)	
BCS	9,982 (88.4)	529 (76.1)	9,453 (89.3)		9,072 (88.8)	322 (75.6)	8,750 (89.4)	
Mastectomy	1,134 (10.0)	157 (22.6)	977 (9.2)		987 (9.7)	99 (23.2)	888 (9.1)	
**Tumor location**				0.800				0.220
Left	5,438 (48.2)	350 (50.4)	5,088 (48.0)		4,922 (48.2)	224 (52.6)	4,698 (48.0)	
Right	5,259 (46.6)	313 (45.0)	4,946 (46.7)		4,782 (46.8)	189 (44.4)	4,593 (46.9)	
Both	589 (5.2)	32 (4.6)	557 (5.3)		511 (5.0)	13 (3.1)	498 (5.1)	
**Tumor location index**				0.794				0.459
Single	10,697 (94.8)	663 (95.4)	10,034 (94.7)		9,704 (95.0)	413 (96.9)	9,291 (94.9)	
Bilateral & synchronous	388 (3.4)	24 (3.5)	364 (3.4)		332 (3.3)	9 (2.1)	323 (3.3)	
Bilateral & metachronous	201 (1.8)	8 (1.2)	193 (1.8)		179 (1.8)	4 (0.9)	175 (1.8)	
Bilateral				0.460				0.360
No	10,863 (96.3)	675 (97.1)	10,188 (96.2)		9,846 (96.4)	416 (97.7)	9,430 (96.3)	
Yes	423 (3.7)	20 (2.9)	403 (3.8)		369 (3.6)	10 (2.3)	359 (3.7)	
**Occurrence in other organs**				<0.001	-	-	-	-
No	10,215 (90.5)	426 (61.3)	9,789 (92.4)		-	-	-	-
Yes	1,071 (9.5)	269 (38.7)	802 (7.6)		-	-	-	-
**Histologic grade**				<0.001				<0.001
1	1,214 (10.8)	32 (4.6)	1,182 (11.2)		1,134 (11.1)	24 (5.6)	1,110 (11.3)	
2	5,522 (48.9)	246 (35.4)	5,276 (49.8)		5,044 (49.4)	153 (35.9)	4,891 (50.0)	
3	4,550 (40.3)	417 (60.0)	4,133 (39.0)		4,037 (39.5)	249 (58.5)	3,788 (38.7)	
**p53(%)**	28.4±23.4	33.4±26.5	28.1±23.1	<0.001	28.3±23.3	33.1±26.2	28.1±23.2	<0.001
**Ki-67(%)**	25.8±21.3	33.1±26.5	25.3±20.8	<0.001	25.4±21.1	32.7±26.3	25.1±20.7	<0.001
**T stage**				<0.001				<0.001
0	602 (5.3)	17 (2.4)	585 (5.5)		556 (5.4)	11 (2.6)	545 (5.6)	
1	7,097 (62.9)	278 (40.0)	6,819 (64.4)		6,521 (63.8)	166 (39.0)	6,355 (64.9)	
2	3,290 (29.2)	316 (45.5)	2,974 (28.1)		2,898 (28.4)	196 (46.0)	2,702 (27.6)	
3	248 (2.2)	58 (8.3)	190 (1.8)		201 (2.0)	33 (7.7)	168 (1.7)	
4	49 (0.4)	26 (3.7)	23 (0.2)		39 (0.4)	20 (4.7)	19 (0.2)	
**N stage**				<0.001				<0.001
0	8,141 (72.1)	315 (45.3)	7,826 (73.9)		7,489 (73.3)	199 (46.7)	7,290 (74.5)	
1	2,461 (21.8)	213 (30.6)	2,248 (21.2)		2,181 (21.4)	131 (30.8)	2,050 (20.9)	
2	516 (4.6)	110 (15.8)	406 (3.8)		417 (4.1)	61 (14.3)	356 (3.6)	
3	168 (1.5)	57 (8.2)	111 (1.0)		128 (1.3)	35 (8.2)	93 (1.0)	

**Tumor subtype**				<0.001				<0.001
Luminal A	7,778 (68.9)	438 (63.0)	7,340 (69.3)		7,063 (69.1)	260 (61.0)	6,803 (69.5)	
Luminal B	1,409 (12.5)	96 (13.8)	1,313 (12.4)		1,249 (12.2)	52 (12.2)	1,197 (12.2)	
Basal	1,207 (10.7)	112 (16.1)	1,095 (10.3)		1,089 (10.7)	83 (19.5)	1,006 (10.3)	
HER2 overexpressing	892 (7.9)	49 (7.1)	843 (8.0)		814 (8.0)	31 (7.3)	783 (8.0)	
**Radiation treatment for curative**				<0.001				<0.001
Yes	8,666 (76.8)	428 (61.6)	8,238 (77.8)		7,843 (76.8)	248 (58.2)	7,595 (77.6)	
No	2,620 (23.2)	267 (38.4)	2,353 (22.2)		2,372 (23.2)	178 (41.8)	2,194 (22.4)	
**Chemotherapy**				<0.001				<0.001
None	874 (7.7)	45 (6.5)	829 (7.8)		835 (8.2)	39 (9.2)	796 (8.1)	
Adjuvant	8,771 (77.7)	412 (59.3)	8,359 (78.9)		8,015 (78.5)	260 (61.0)	7,755 (79.2)	
Neoadjuvant	1,641 (14.6)	238 (34.2)	1,403 (13.2)		1,365 (13.4)	127 (29.8)	1,238 (12.6)	
**Anti HER2**				<0.001				<0.001
No	9,482 (84.0)	479 (68.9)	9,003 (85.0)		8,758 (85.7)	334 (78.4)	8,424 (86.1)	
Yes	1,804 (16.0)	216 (31.1)	1,588 (15.0)		1,457 (14.3)	92 (21.6)	1,365 (13.9)	
**Anti hormone**				<0.001				<0.001
No	2,428 (21.5)	210 (30.2)	2,218 (20.9)		2,209 (21.6)	150 (35.2)	2,059 (21.0)	
Yes	8,858 (78.5)	485 (69.8)	8,373 (79.1)		8,006 (78.4)	276 (64.8)	7,730 (79.0)	

BMI, body mass index; WBC, white blood cell; BCS, breast conserving surgery; HER2, Human epidermal growth factor receptor 2.
