# Supplementary figures and images for: Mortality Prediction Modeling for Patients with Breast Cancer Based on Explainable Machine Learning
Source: Cancers (Basel). 2024 Nov 12;16(22):3799. doi: 10.3390/cancers16223799 (PMC11592669; doi:10.3390/cancers16223799)

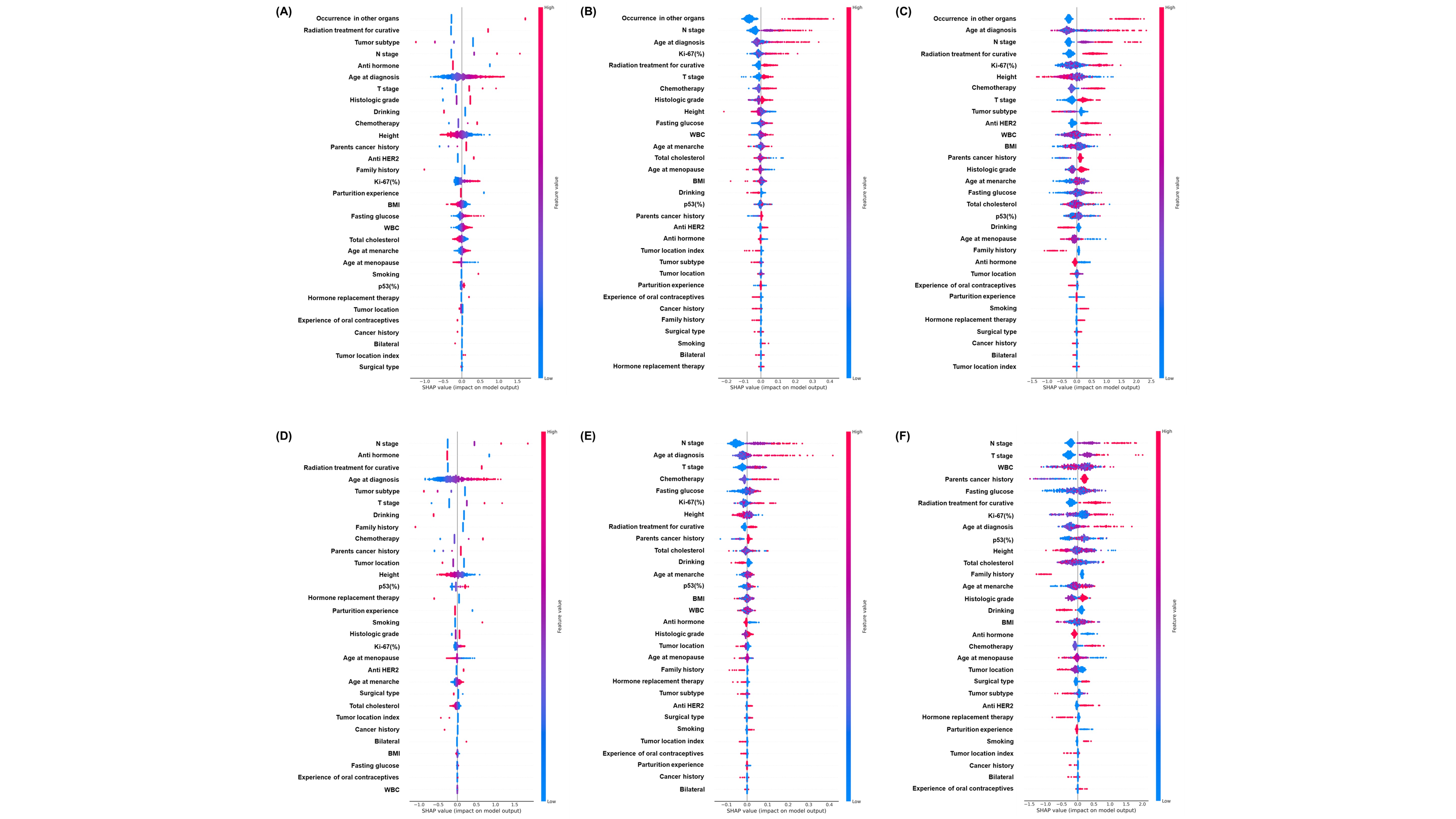

Supplement: Supplementary file 1 [file cancers-16-03799-s001.zip › FigureS1.jpg]

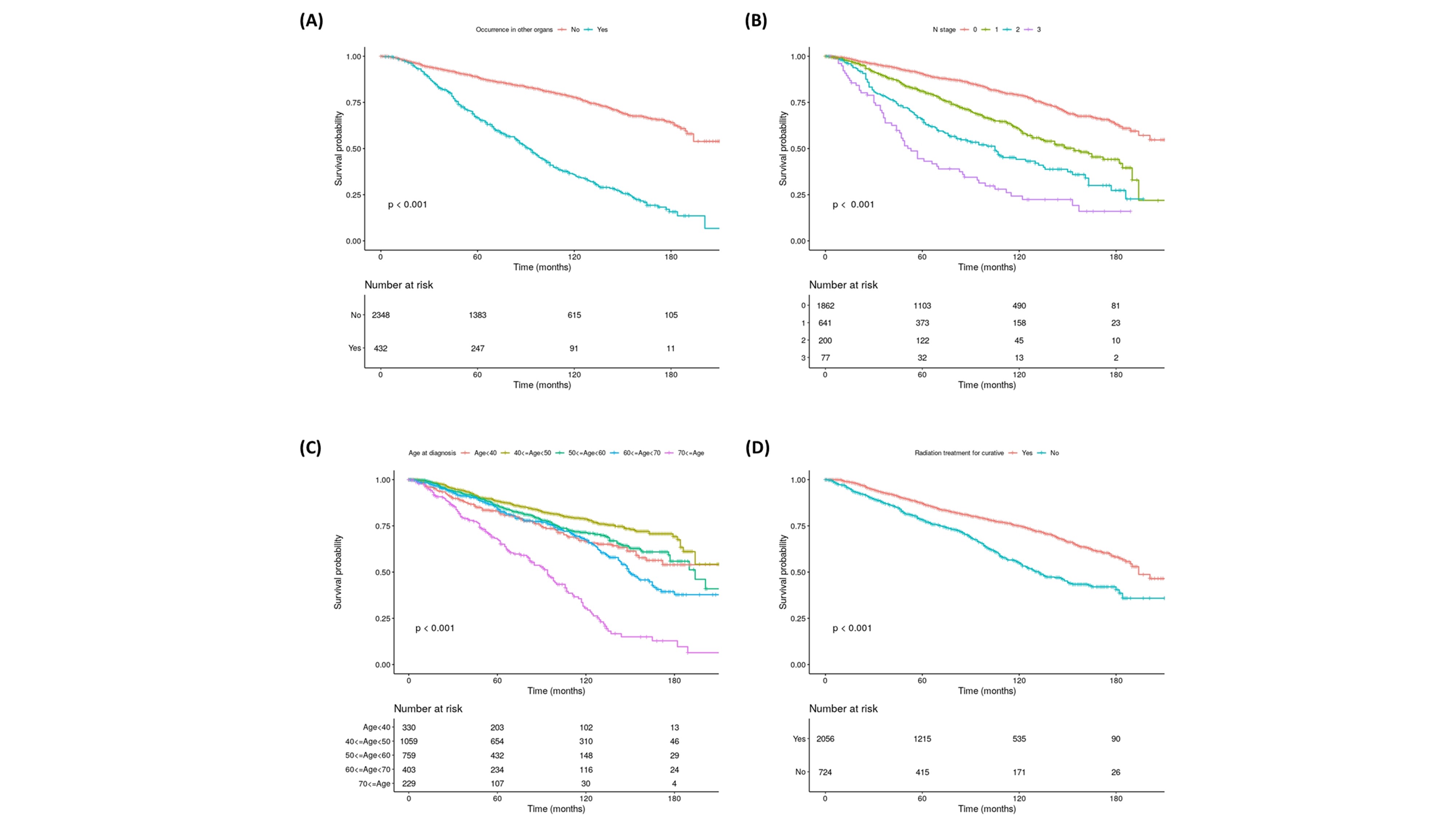

Supplement: Supplementary file 1 [file cancers-16-03799-s001.zip › FigureS2.jpg]

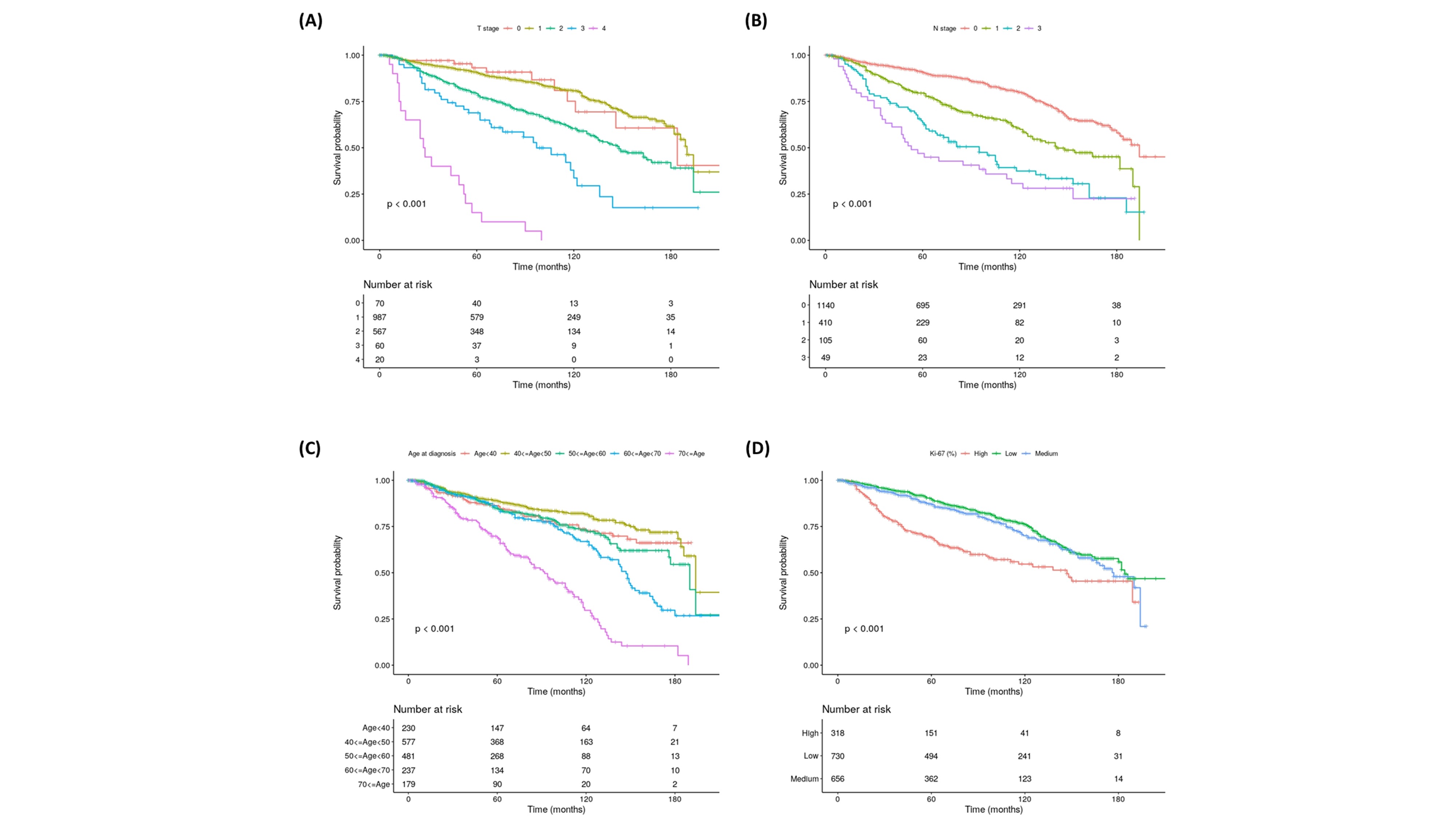

Supplement: Supplementary file 1 [file cancers-16-03799-s001.zip › FigureS3.jpg]

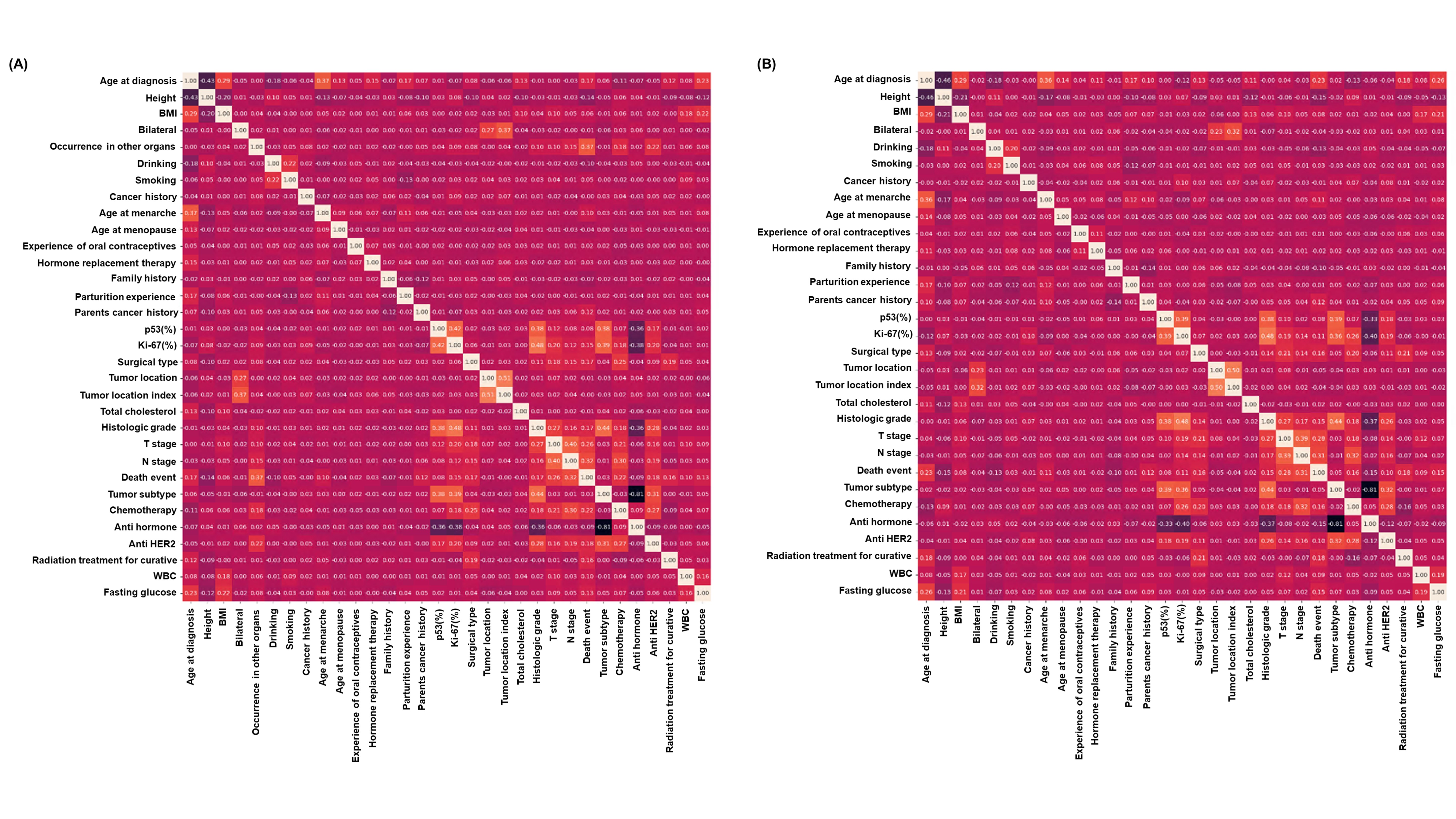

Supplement: Supplementary file 1 [file cancers-16-03799-s001.zip › FigureS4.jpg]

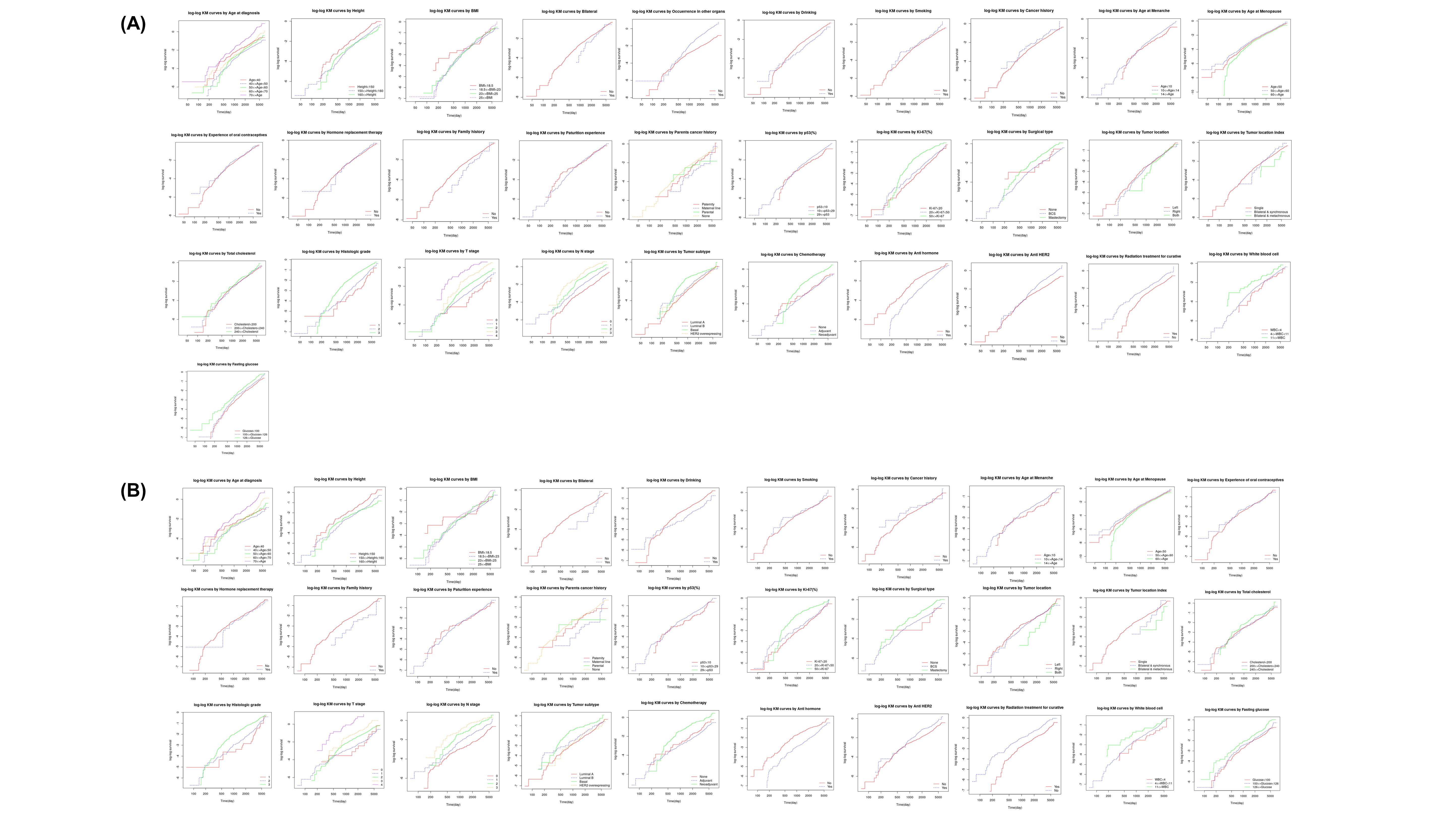

Supplement: Supplementary file 1 [file cancers-16-03799-s001.zip › FigureS5.jpg]
